# Positioning Shifts From Told Self to Performative Self in Psychotherapy

**DOI:** 10.3389/fpsyg.2020.572436

**Published:** 2020-10-26

**Authors:** Arnulf Deppermann, Carl Eduard Scheidt, Anja Stukenbrock

**Affiliations:** ^1^Pragmatics Department, Leibniz-Institute for the German Language, Mannheim, Germany; ^2^Department of Finnish, Finno-Ugrian and Scandinavian Studies, University of Helsinki, Helsinki, Finland; ^3^Department of Psychosomatic Medicine and Psychotherapy, Faculty of Medicine, Albert-Ludwigs-Universität Freiburg, Freiburg, Germany; ^4^Faculté des Lettres, Section d'allemand, Université de Lausanne, Lausanne, Switzerland

**Keywords:** psychoanalysis, conversation analysis, positioning, interpretation, psychotherapy, social interaction, self

## Abstract

According to Positioning Theory, participants in narrative interaction can position themselves on a representational level concerning the autobiographical, told self, and a performative level concerning the interactive and emotional self of the tellers. The performative self is usually much harder to pin down, because it is a non-propositional, enacted self. In contrast to everyday interaction, psychotherapists regularly topicalize the performative self explicitly. In our paper, we study how therapists respond to clients' narratives by interpretations of the client's conduct, shifting from the autobiographical identity of the told self, which is the focus of the client's story, to the present performative self of the client. Drawing on video recordings from three psychodynamic therapies (*tiefenpsychologisch fundierte Psychotherapie*) with 25 sessions each, we will analyze in detail five extracts of therapists' shifts from the representational to the performative self. We highlight four findings:

• Whereas, clients' narratives often serve to support identity claims in terms of personal psychological and moral characteristics, therapists rather tend to focus on clients' feelings, motives, current behavior, and ways of interacting.

• In response to clients' stories, therapists first show empathy and confirm clients' accounts, before shifting to clients' performative self.

• Therapists ground the shift to clients' performative self by references to clients' observable behavior.

• Therapists do not simply expect affiliation with their views on clients' performative self. Rather, they use such shifts to promote the clients' self-exploration. Yet, if clients resist to explore their selves in more detail, therapists more explicitly ascribe motives and feelings that clients do not seem to be aware of. The shift in positioning levels thus seems to have a preparatory function for engendering therapeutic insights.

## Introduction

The self is far from being a unified notion (e.g., Neisser, 1988). This holds also true for different facets of the self, which may be at issue in social interaction. As Bamberg (1997), Lucius-Hoene and Deppermann (2004), Bamberg and Georgakopoulou (2008), and Deppermann (2015) have argued, positioning of selves in narrative interaction can occur on at least two levels: A representational level concerning the autobiographical, told self, and a performative level concerning the interactive and emotional self of the teller. The performative self usually is much harder to pin down, because it is non-propositional and enacted. In everyday interaction, it is unusual to explicitly describe aspects of the partner's performative self. Psychotherapy is different: in their interpretations, therapists regularly topicalize aspects of the performative self explicitly, in particular those that the client does not seem to be aware of, but may offer insights into the client's problems. One environment in therapist's responses shift to the client's present performative self are autobiographical narratives by the client that serve to support a certain identity claim made by the client.

Such shifts are sensitive moments in the therapy. They imply that the therapist claims epistemic authority concerning the client's current feelings, motives, or the interpretation of their behavior. This is in contrast to the usual assumption in Western cultures that the subject has privileged access to the self (Heritage, 2011; Gertler, 2020). Drawing on video recordings from three psychodynamic therapies (*tiefenpsychologisch fundierte Psychotherapie*) with 25 sessions each, in this paper, we analyze five extracts in which the therapist's interpretation shifts to the performative self of the client in response to a client's story. We first introduce Positioning Theory as an approach to conceptualize and study the self in (narrative) interaction on the basis of audio and video recordings (section Positioning in Narrative Interaction). Section Client's Self-Positioning and Therapist's Shifts to the Performative Self lays out the generic sequential structure of episodes in psychotherapy in which therapists shift from clients' autobiographical narratives to their performative self, including the ensuing negotiation of therapists' interpretations by both parties. After a description of data and methods used in this study (section Data and Methods), the main body of the paper is devoted to the in-depth analysis of five extracts in which therapists shift to the client's performative self in their sequential context (section Shifts to the Performative Self in Psychotherapy: Five Exemplary Cases). Section Conclusion summarizes and discusses the findings with respect to their import for psychodynamic therapy.

## Positioning in Narrative Interaction

Narratives are the primary mode of self-reference in psychodynamic therapy (Boothe, 2004, 2010). Clients tell biographical episodes, recent events, dreams, etc. Yet, already early on, the psychoanalytic talking cure has involved not only recollection but also the focus on the client's repetition of entrenched behavioral patterns in the therapeutic situation (Freud, 1924[1914]). More recent approaches to psychoanalysis highlight this interactive dimension of psychotherapy as being crucial for change (Streeck, 2004). Well-known psychoanalytic phenomena like resistance, transference, and counter-transference operate mainly on the interactional level (Greenson, 1978). Yet, in modern versions of psychoanalysis, this highly asymmetric understanding of the psychotherapeutic relationship, which presupposes a knowing analyst vs. a client who is unaware of their psychodynamic motives, is replaced by a more symmetrical understanding of an intersubjective field to which both client and analyst are equally contributing. Interpretation in the post-bionian model of the analytic field “is no longer considered as the expression of the analyst's knowledge about the client, but as a multidimensional offer of meaning intended to bring new ideas and emotions to life at an intersubjective level” (Civitarese, 2020).

In this paper, we draw on Positioning Theory for the analysis of different facets of the self that are treated as relevant in psychotherapeutic interactions. Bamberg (1997) and Bamberg and Georgakopoulou (2008) distinguish between level-1 positioning, involving the self “as a character in the story” (Bamberg and Georgakopoulou, 2008: 380), and level-2 positioning, the way in which the teller “positions himself (and is positioned) within in the interactive situation” (Bamberg and Georgakopoulou, 2008: 385). Lucius-Hoene and Deppermann (2004) and Deppermann (2015) have elaborated this model, distinguishing representational positioning of the autobiographical, told self, and performative positioning of the emotional and interactive self. Performative positioning is implicit and importantly includes bodily displays. Both modes of positioning are manifested by different discursive practices:

- Representational positioning of the told self includes
  ° description of actions, feelings, thoughts, intentions, etc. by narrative clauses;
  ° ascriptions to present and past self;
  ° enactment by reported dialogue;
  ° reported statements by third parties;
  ° metanarrative comments on and categorizations from past or present point of view;

- Performative positioning of the emotional and interactive self includes
  ° claims to facets of identity by narrative performance and interactional conduct;
  ° affective displays by prosody, facial expression, gaze, etc.;
  ° positioning vis-à-vis the interlocutor.

Positioning involves not only self-positioning but also other-positioning, i.e., the ascription of facets of identity to the interlocutor (or third parties), which can also be done both by explicit representations or in a performative mode. Self- and other-positioning can imply each other, as, e.g., when adopting performatively the role of a teacher, the addressee is other-positioned as a student.

Positioning Theory seeks for an analysis of identities as they become referred to and indexed in narrative talk-in-interaction. Conversation Analysis (Schegloff, 2007) equips us with the methodology of sequential analysis, which is needed to show how participants observably orient to situated facets of identity in their interactions and how they understand, treat, and negotiate identity displays. Positioning Theory is compatible with conversation analytic views on the self in interaction (Wilkinson and Kitzinger, 2003). It encompasses membership categorization (Jayyusi, 1984), but it can also address how participants recognizably orient to moral concerns of the self, i.e., the participant's face (Goffman, 1955). Yet, the Positioning approach goes beyond these two approaches by attending to the biographical and to the psychological dimensions of the self as well as they become manifest in interactional episodes (e.g., bodily self-perception, reflexive self-positioning, and ascription of feelings and motives; Deppermann, 2013).

## Client's Self-Positioning and Therapist's Shifts to the Performative Self

Our study deals with narratives in psychotherapy that clients themselves interpret in terms of their personal identity. Thus, we deal with sequences of interaction in which the self is undoubtedly in focus for the participants. In the same way as Vehviläinen (2003) and Voutilainen et al. (2010) have shown, therapists in our data mostly initially respond with empathy or partial agreement with the client's identity claim. However, they never respond with unrestrained agreement or displays of reciprocity, e.g., by a second story about own experiences, as has been shown in other interaction types by Heritage (2011) and Kupetz (2016), but which would violate the neutrality requirement of the psychoanalytic technique. Instead, they provide candidate understandings (Weiste and Peräkylä, 2013), which may even be challenging (Antaki, 2012), and continue their turns by producing interpretations that attribute unconscious motives or unavowed feelings to the client (Greenson, 1978: 37–45; Peräkylä, 2008; Weiste et al., 2015).

The type of sequences we will discuss has the following shape:

- 1A: Story-telling: Client (CL) tells autobiographical story.
1B: Identity claim: CL interprets story in terms of identity ascription.
- 2A: Display of empathy: Therapist (TH) provides display of empathy and/or partial agreement.
2B: Interpretation: Therapist shifts focus to the client's present, performative self, pointing out identity aspects that contrast with, undermine, or reframe the client's self-ascription. This shift to the performative level is brought about by focusing on the client's present behavior, their feelings (Peräkylä, 2008; Voutilainen et al., 2010), and/or the (unconscious) motives that make the client tell their story. The focus on the client's performative self can combine with a focus on how the client manages the relationship with the therapist.
- 3: Negotiation of the interpretation: Therapist and client negotiate the meaning or the validity of the therapist's interpretation (Peräkylä, 2005, 2010). This part will minimally consist of the client's response to the therapist's intervention 2B, but may extend to a larger negotiation of interpretations and ascriptions to the client; it can also include argumentative and narrative elaborations.

In this paper, we examine (1) how clients in psychodynamic psychotherapy interpret their own autobiographical narratives in terms of who they are (Bamberg, 2011), (2) how therapists respond to narratives by interpretations shifting to clients' performative self, and (3) how both parties negotiate the meaning and the validity of the therapist's interpretation of the client's self, focusing on whether both parties manage to arrive at a shared understanding of the client's identity, motives, and feelings.

## Data and Methods

We draw from two psychodynamic focal therapies with 25 sessions each, video-taped at the Medical Faculty of the University of Freiburg, Center for Psychiatry, Psychosomatic Medicine and Psychotherapy in 2017–2018. Excerpts 1–3 from the first therapy include a male client in his late 60's suffering from depression and a functional pain syndrome subsequent to the death of a family member. The young female therapist was still in her analytic training. Excerpts 4 and 5 are from the second therapy with a young woman in her 20's suffering from psychogenic seizures. The therapist was a psychoanalytically trained senior staff member.

All sessions were exhaustively coded for all occurrences of different types of therapists' responses like understanding checks, repetitions, formulations, and interpretations. Fifty-five instances of therapists' interpretations were transcribed according to GAT2 (Selting et al., 2011, with selected additional multimodal annotations, Mondada, 2018; see Appendices A, B) together with their sequential context (with a duration between 1:59 and 7:01 min), i.e., preceding client's narratives and descriptions and following negotiations of the interpretation. All 55 extracts were analyzed by the three authors together using Conversation Analysis with a focus on sequential organization and turn design. Among the 55 extracts, 5 extracts (out of 10) in which the therapist shifts to the performative self of the client have been chosen for this paper.

## Shifts to the Performative Self in Psychotherapy: Five Exemplary Cases

In the following, we analyze in depth five extracts in which therapists respond to clients' autobiographical self-positioning by shifting to performative aspects of client's current self. We have chosen extracts that give evidence of the various dimensions of the performative self that therapists address in their interpretations:

- Unconscious motives that inform client's storytelling (section Redefinition of the Motives for Storytelling: From Identity-Display to Defense Mechanism),
- emotions that are in contrast to client's explicit self-presentation (section Inference From Presuppositions in the Client's Story: From Rational Self-Presentation to Emotional Distress),
- the client's way to conduct the interaction with the therapist (section Interpreting the Client's Way of Designing the Psychotherapeutic Relationship: Claiming an Analogy Between Agentive Self-Relationship and Interpersonal Relationship),
- different objects or causes for the client's emotion (section Observing Non-verbal Conduct: Focusing on an Emotion and Shifting its Object),
- a challenge of the authenticity of the client's representational self-positioning (section Summarizing Impressions From Client's Talk: Challenging the Authenticity of the Performance).

Each section closes with a conclusion concerning the positions that the participants accomplish with respect to the client's self in the extracts: client's representational self-positioning is summarized and related to how the therapist other-positions the patient by shifting to the client's performative self.

### Redefinition of the Motives for Storytelling: From Identity Display to Defense Mechanism

Extracts 1–3 are from the first therapy with a young female therapist and an elderly male client. One of the recurrent topics of the sessions are CL's conflicts with authority figures, which had also been discussed before extract 1 starts. The client tells a story about a conflict with a superior at work as evidence for how he acquired psychological strength. The therapists shift to the motives for the client's storytelling^1^.

**Extract 1:** Therapy_I_12_44:10-48:40.

001	CL	da ischt dann an diesem meine STÄRke entstande;
		*there then at this my strength developed*
002		(0.54)
003	CL	an dEm TAG,
		*on that day*
004	TH	[HM_hm–]
005	CL	(.) wo sie_s jetzt mir dann geSAGT [hat,=]
		*where she now said it to me then*
006		=also DIEter,
		*well NAME-CL*
007		(.) staTIONsleitung,
		*position of the head nurse*
008		(0.69)
009	CL	kriegt die Ulli?
		*will be assigned to NAME-colleague*
010		(0.14)
011	TH	HM_hm,
012		(0.62)
013	CL	und es wär LIEB,
		*and it would be kind*
014		(0.13)
015	CL	und k
		*and*
016		(0.18)
017	CL	tOll von dir wenn du sie EINarbeiten würdsch,
		*great if you introduce her to the work*
018		(0.29)
019	CL	un sie k äh als stEllvertr[etung weiter] hin ARbeiten würdscht.
		*and will carry on working as vice head*
020	TH	[HM_hm,]
021		(0.38)
022	CL	((lipsmack)) un ich gesagt liebe SILke,
		*((lipsmack)) and I said dear name-superior*
023		(1.11)
024	CL	das WAR_s.
		*that was it*
025		(0.56)
026	TH	hm_HM,
027		(0.27)
028	CL	SO: (0.34) lass (0.12) ich (.) nIcht (.) mit mir (.) UMgehen.
		*I won*'t let me be treated that way**
029		(0.64)
030	CL	ich hab (0.15) äh drEI viertel jahr die staTION geführt;
		*I have led the ward for nine months*
031		(0.46)
032	CL	top.
		*perfect*
033		(0.84)
034	CL	ja?
		*yes*
035		(0.12)
036	CL	[da gab_s] (.) KEIne beschwerde;
		*there hasn‘t been any complaint*
037	TH	[HM_hm;]
038		(0.23)
(…)		
102	CL	un NE,=
		*and no*
103		=ich hab noch zu ihr geSAGT;=
		*I said to her still*
104		=äh SILke,=
		*erm name-superior*
105		=ich nehm jetz meinen RESCHTurlaub,
		*I now take my residual leave*
106		(0.52)
107	CL	und dann äh äh äh wirscht du mir nicht mehr SEHen.
		*and then erm you won't see me again*
108		(0.1)
109	TH	HM_hm;
110	CL	ja du KANNscht aber net einfach so:-
		*well but you cannot simply like this*
111		(0.36)
112	CL	und da hat aber diese SCHULleitung;
		*and there this director of the nursing school*
113		(0.24)
114	CL	der hat mir dann gHOL[fe,]
		*he helped me then*
115	TH	[HM]_hm,
116		(0.21)
117	CL	und hat mir dann quasi
		kein KÜNdigungsvertrag;=
		*and then did not (give) me like a cancelling contract*
118		=sondern so_ne (0.31) überBRÜCK[u:ng;]
		*but kinda bridging*
119	TH	[HM]_hm;
120		(0.25)
121	CL	< < decr> und konnt [ich als sch]ulassistent anfangen >.
		*and I could start as a school assistant*
122	TH	[ja:,]
123		(0.7)
124	TH	hm^*^:–
	cl	^*^puts hand on his chest—->
125		(1.1)^*^
	Cl	—->^*^
126	CL	es [koscht] mich immer noch;=
		*it still costs me*
127	TH	[hm;]
128	CL	=^*^aber TRAUT hab ich mich;
		*but I dared to*
	cl	^*^smiles slightly———->
129		(0.5)
130	TH	< < p> haben sich DURCH^*^gesetzt;>
		*(you) prevailed*
	cl	———————————————————————————^*^
131	CL	< < p> hab mich DURCHgesetzt>;
		*(I) prevailed*
132		(1.2)
133	TH	auf mal so_ner ganz AN:deren ebene;=
		*just on a very different level*
134		=also_s THE:ma,=
		*so the topic*
135		=mit dem wir ja ANgefangen haben die stunde;=
		*with which y‘know we have started this session*
136		=war ja (.) eben der neuroLOge?
		*was y'know PTCL the neurologist*
137		((lipsmack))
138	CL	hm_[HM,]
139	TH	[äh:]m (.) der die rolle sp
		*erm who pl- the role*
140		(0.13)
141		TH ((creak))
142		(0.16)
143	TH	der so: MACHT noch hat so in ihren gedanken,
		*who still has kinda power like in your thoughts*
144		(0.5)
145	TH	ah:m; (0.6)
		*erm*
146	TH	wo sie sich NICHT durchsetzen konnten.
		*where you did not prevail*
147	TH	(.) d[er irgendw]ie:-
		*who somehow*
148	CL	[hm_HM.]
149		TH °h (.) un jetz haben sie mir (.) ganz viele geSCHICHten
		erzählt?=
		*and now you have told me a whole lot of stories*
150		=von denen die [ganze STUNde] über,
		*of which the whole session long*
151	CL	[wo des gekLAPpt] hat,
		*where it worked*
152	TH	°h (.) geNAU.
		*exactly*
153		(0.24)
154	TH	°h°
155	CL	ja–
		*yes*
156	TH	(.) und
		*and*
157		(1.15)
158		TH ich frag mich ist das so_n mechaNISmus von ihnen;=
		*I wonder is this kinda mechanism of you*
159		=dass sie sich dann dA dran erINnern?
		*that you remember that then*
160		°h ((lipsmack)) u:m (.) sich so !SE:LBST! (.) äh:m (0.21) zu zei:
		*((lipsmack)) erm in order to erm sho- yourself this way*
161		(.) also nochmal AUFzuzeigen:;=
		*I mean display once again*
162		=da gIbt_s auch situationen wo ich das meistern KONnte?
		*there are also*situations in which I (= client) could cope with that**
163		(0.53)
164		damit sie sich weniger SCHWACH fühlen?=
		*so that you feel less weak*
165		=in den ANderen situationen;=
		*in the other situations*
166	TH	=so was ist (0.64) die ROLle (.) dieser geschichten und dieses
167		(.) dieses erzählens [da(bei),]
		*so what is the role of these stories and this this telling there*
168	CL	[das isch_ne] des
		isch_ne SAche,
		*this is a this is a thing*
169	CL	(0.4) da gebe sie mir jetz en SCHLÜSsel?
		*there you now give me a key*
170		(0.44)
171		TH hm_HM,
172	CL	(0.2) weil sie des jetz SAge,
		*because you say this now*
173		(0.6)
174	CL	des HILFT mir,
		*this helps me*
175		(0.35)
176	CL	weil dann werde ich in ZUkunft,=
		*because then I will in the future*
177		=wenn da mal wieder irgendwie was ISCH oder so,
		*if there is just again something or so*
178	CL	°h (.) werde ich mich (.) ÖFter mal an diese situationen erINnern.
		*I will just remember these situ ations more often*
179		(0.52)
180		TH °h [°h]
181	CL	[wo isch] STARK war;
		*when I was strong*
182		(0.73)
183	TH	ich hab das nich ich ich [((knarrt))]
		*I have that not I ((creak))*
184	CL	[NE des:]
		*no this*
185		ich glaube ich wollte jetz erstmal nur so (.) in FRAge stellen.=
		*I guess I wanted now first just only kinda question*
186		=was sie damit MACHen;=
		*what you do with them (= the stories)*
187		=ich hab jetzt
		*I have now*
188		°h (.) ich !WEISS! nicht ob es die passende strateGIE [is.]
		*I don‘t know if it is the proper strategy*
189	CL	[hm]_HM–
190	TH	°h so_s war einfach nur_ne FRA:ge von dem (.) was hier
		pasSIE:RT.=oder?
		*like it was simply only a question about what is happening here, right?*

#### Client Tells a Story of Prevailing Over an Authority Figure and Interprets it in Terms of Strength (001–131)

The story preface: (“my strength developed on that day,” “da ischt an diesem meine STÄRke entstande; an dEm TAG,” 001-003) projects an autobiographical key narrative. It is a story about pride and self-assertiveness when facing a threat of devaluation: The client considers himself to be treated as unfair and disrespectful, because he is not appointed as a ward nurse, although he fulfilled this position for 9 months perfectly well and without any complaints (30–36), was praised by the doctors and proved to be a responsible and strict leader of his team (between 38 and 102, not shown). The climax of his story is a reported interaction with his superior, in which he rejects the request to introduce the new ward nurse to her work; instead, he cancels his job on the spot (103–110) and changes to another position that was offered to him in a neighboring nursing school (112–121). The final morale frames it as a story about courage (124–128) in the face of an unjust authority, who does not respect him.

#### The Therapist's Response: Inquiring Into the Motivation for Telling Stories of Strength

As a first reaction, the therapist shows understanding and empathy by collaboratively formulating the gist of the story as “you prevailed” (130), which is confirmed by the client's repeat (131). The therapist then announces to switch to a “completely different level” (133). She refers back to the stories that the client told over the course of the sessions (135–152) and puts forth the hypothesis that the client tells stories of strength in order to fight feelings of weakness (149–165). In psychoanalytic terms, the client's stories are interpreted as a variety of the defense “mechanism” (158) of reaction formation (Freud, 1937). This can be conceived of as resistance against facing the painful feeling of being left out. The categorization as a “mechanism” (158) of the client implies that there is a motivational process the client is not aware of. The therapist's intervention is clearly not affiliative. It treats the kind of stories the client tells as a behavior in need of psychological analysis. However, the psychoanalytic term “mechanism” may not be transparent to the client, who therefore misses that it hints at questionable motives in need of further exploration (see step 3).

#### Ensuing Negotiation: Useful Self-Management Strategy vs. Object of Motivational Inquiry

The client does not take the therapist's intervention up as a cue for questioning his hidden motives. Instead, he treats it as a recommendation of a self-management technique to fortify his identity when he feels shaken (168–181). The therapist disclaims that it was her intention to recommend using stories of strength as a self-management technique. By his third-position repair (Schegloff, 1992), she rejects the client's response as resting on a misunderstanding of her interpretation; instead, she insists on questioning the use of this strategy (183–190).

##### Self- and other-positioning in extract 1

In Extract 1, the client positions his told self mirrored by the perspectives of third parties: He is treated disrespectfully by superior vs. praised by doctors and supported by the head of the nursing school. Third parties serve as warrant of his entitlement to the position he is denied and to his moral rights against his superior, who disrespects him. The client explicitly claims and narratively displays an identity of strength, which he has acquired by a courageous act. The story is a biographical key story, which describes a change in the client's identity by his own agency against all odds and which establishes an important link between the client's former and his present self. The story is presented as a warrant for the factuality of his identity claim: the representational level of the story is treated as primary. The client positions himself by performance as well: His past self is re-enacted with syntonous affect (indignation, anger), and he displays pride by smiling when formulating the morale of the story. Interactively, the client calls for recognition of his courage and his achievement, inviting affiliation with his identity claim, maybe even fishing for the therapist's praise.

The therapist displays empathy with the client and his claimed self of strength. Nevertheless, she shifts the focus and treats the performative level as primary by interpreting the client's story not as a factual story about the becoming of his identity (= representational positioning), but as a performative, strategic self-presentation, whose function is to be questioned, because it serves to avoid facing experiences and feelings of weakness.

The levels of positioning that client and therapist treat as being focal are at clash. While the client focuses on the biographical becoming of his identity, the therapist treats the motivation for claiming this identity as more important. Yet, the client does not understand the therapist's intervention as a cue to explore his motivations in more detail. Instead, he takes it up as a recommendation, which supports the psychological usefulness of his identity claim as a means to enhance his agency and well-being.

### Inference From Presuppositions in the Client's Story: From Rational Self-Presentation to Emotional Distress

In extract 2, the therapist's interpretation focuses on an emotion (sadness) that contradicts the client's overt claim (acceptance) but is treated as being presupposed by the client's word choice. Already in the first session, the therapist and the client talk about his loss of bodily strength and endurance because of aging. Comparing himself to others who are worse off, the client stated: “Ich muss zuFRIEde sein.” (“I must be satisfied,” 05:54). Afterwards, the client told about the need to be cautious because of problems with his prostate gland. After the client concludes his story with an interjection that expresses concern (“HA:I yai yai,” 03), the therapist refocuses on the client's prior claim that he has to be satisfied with his health condition, and casts it into doubt (13–15).

**Extract 2:** Therapy I_1_09:07-10:46.

01	CL	aber des_sin Immer noch diese (1.0) diese gSCHICHte;
		*But these are still always these these stories*
02		(0.3)
03	CL	die mir (1.04) dann sage HA:I yai yai,
		*which then tell me ((interjections))*
04		(3.7)
05	TH	< < h>HM_hm,>
06		(1.3)
07	TH	sie haben Eben gesagt; (0.2)
		*you just said*
08	TH	ich !MUSS! (.) zufrieden sein.
		*I must be satisfied*
10		(1.0)
11	TH	mit dem (0.9) mit ihrer fitness mit SIEBzig.
		*with the with your fitness being seventy*
12		(1.8)
13	TH	dieses !MUSS! (.) klingt so ein bisschen so^*^:-
		*This must sounds like kinda little bit as if*
	cl	^*^smiles—>
14		(1.0)
15	TH	so !GANZ! stehen sie noch nich daH[INter;]
		*as if you are not fully behind it*
16	CL	[nee:,]
		*no*
17	CL	((laughs))
18		(2.7)
19	CL	ja ich würd scho
		*well I would PRT*
20		(0.3)
21	CL	((clears throat))
22		(1.0)
23	CL	ich freue mich irgendwie jetz auf den FRÜHling,=
		*I am looking forward to spring now somehow*
24		=und hab halt schon un äh des gFÜHL, =^*^
		*and (I) somehow have erm the feeling*
	cl	—————————————————————————–^*^
25		=dass ich äh: auch wenn ich jetz auf_n HIRZberg äh äh gehe würd;
		*that I erm even if I now would walk up the NAME-OF-MOUNTAIN*
26		(0.6) ah: (.) oder irgendwo ANdersch,
		*erm or somewhere else*
27		(0.6) da ein steiler BERG (.) bei uns,
		*there a steep mountain in our region*
28		(0.5) äh da dass ich ((knarrt)) das mit dem:
		*erm there that I ((creaks)) the thing with the*
29		ah: mit der (0.3) i es GE,
		*erm with the sacroiliac joint*
30		(0.3)
31	TH	HM_[hm,]
32	CL	[sehr wahr]scheinlich (.) ah:: (1.3)
		*very probably erm*
		proBLEme kriegen [würde.]
		*(I) would run into trouble*
33	TH	[HM_hm,]
34		(0.66)
35	CL	des isch_s des geFÜHL isch [da:]
		*this is it the feeling is there*
36	TH	[HM_]hm,
37		(1.5)
38	TH	genau des_so diese verNÜNftige seite die zu ihnen sagt,
		*exactly there is this rational side which says to you*
39		(2.2)
40	CL	[isch s]o_ne ambivaLENZ;
		*(it) is kind of an ambivalence*
41	TH	[aber]
		*but*
42	TH	geNAU.=
		*exactly*
43	TH	=was mich aber interessiert is so eben auch (.) die ANdere;=
		*but what interests me is just also the other*
44	TH	=so_n bisschen die emotioNAle,=
		*kinda little bit the emotional*
45	TH	=vielleicht auch die TRAUerseite;
		*perhaps also the sadness-side*
46	CL	hm_[HM;]
47	TH	[di]eses ABschiednehmen;
		*this saying goodbye*
48		(0.8)
49	TH	von was was sie ihr ganzes leben (1.5) WICHtig war;=Oder?
		*to something which your whole life was important, right?*
50	CL	((clears throat))(0.4) schon:;
		*well that's true*
51		(1.8)
52	CL	weil: es ISCH halt SO,
		*because it is like this*
53		(0.4) zum beispiel mein BRUoder.
		*for example my brother*
54		(0.8) der hat zwar auch im KNIE:,
		*he has problems with his knee as well*
55		(.) un der un der leidet immer;=
		*and he and he suffers always*
56		=weiß der TEUfel was alles;=
		*the devil knows what else*
57		=muss aber keine medikaMENte un nix nehme;
		*but he does not have to take any drugs or anything*
58		(0.6) aber da: (.) der (.) der kann wieder < < knarrig> äh:> TOUrenschie fahren;
		*but there he he can erm do ski touring again*

#### Client's Story (Before the Extract Until Line 03)

The client talked about health problems, his concerns, the need to be cautious, and his acceptance of his health condition, given that others are worse off.

#### The Therapist's Response: Therapist Doubts That Client Is Satisfied With His Health Condition (05–15)

The therapist quotes the client's earlier claim that he must be satisfied (07–11) and highlights his use of the modal verb “muss” (“must,” 08), which expresses constraint (Zifonun et al., 1997, 1881–1923). She interprets the choice of the verb as making perceivable (“klingt so,” “sounds like,” 13)^2^ that the client is not fully emotionally ready to accept his situation, or, alternatively, not fully convinced that he has to accept it (13–15). This interpretation makes an unavowed emotion explicit (Peräkylä, 2008; Voutilainen et al., 2010) and is clearly designed to achieve intersubjectivity by referring to common ground concerning the client's narrative action in order to persuade him (Weiste et al., 2015). Yet, the therapist thereby changes from the client's explicit self-positioning to an impression she has gained about his present emotional and cognitive stance toward his health condition that the client is taken as not having communicated, but given off (Goffman, 1959) by his linguistic choice.

#### Ensuing Negotiation: Ambivalence Between Desire and Physical Limitations vs. Focus on Sadness About Loss (16–35)

In overlap with the therapist's interpretation, the client starts to smile, agrees (16), and then laughs slightly (17), which seems to index that he feels being understood. He continues by deploying the imaginary scenario of looking forward to climbing a high mountain (what has been his favorite hobby), stating that he would expect to have problems with his sacroiliac joint (17–35). The therapist confirms and categorizes his account as expressing his rational side (38), thus constraining its validity as expressing only one part of the client's self. The client, in turn, concludes that there is an ambivalence (between his desire to do sports and concerns about limitations of his health condition, 40). By marking a shift in perspective (“the other side,” 43), the therapist again changes focus to the client's emotions, now explicitly introducing sadness about having to say farewell to well-beloved habits (43–49). Using the format “what interests me is X” (43), the therapist does not explicitly claim that the client is sad about having to say farewell, nor does she explicitly ask if this is the case. Rather, she establishes this emotional state as a thematic focus (43–49). Using the demonstrative article (“this saying goodbye,” 47), she presupposes that it is relevant for and known by the client, urging him to elaborate with a tag (49; König, 2020). The client grants the therapist's intervention by a concessive particle (50); he expands his turn by a story about his brother, who, in contrast to the client, is still able to practice physically demanding sports, although he suffers from various illnesses as well (54 until beyond the extract). This story of social comparison implicitly avows discontent and the feeling of injustice of not being able to perform any more like significant others of about the same age. The client thus partially aligns with the therapist in exploring his emotions concerning physical limitations further, however, without dealing with the emotion of sadness about loss in particular.

##### Self- and other-positioning in extract 2

The client positions himself as a person who is fond of doing all sorts of physical activities and sports, but who is concerned about his condition because of health problems that have increasingly developed with age. He conceives of himself as being in an ambivalent position, torn between the desire for bodily activities and the acceptance of increasing physical limitations.

While confirming the client's self-positioning and his pragmatic orientations, the therapist focuses on the client being emotionally more affected from the sadness about losses related to aging than he seems to admit. Her first intervention closely builds on the client's prior words and, drawing on a semantic presupposition of his statement, she infers emotional trouble on his part concerning age-related changes in his health condition (07–15). As the client does not take up the issue of exploring the emotions that might cause his “not fully being behind” his acceptance, the therapist explicitly states “sadness about loss” as a topic, an emotion that she at least tentatively attributes to the client.

The explicitation of the inference from the client's linguistic choice is thus a means to cue the client's self-exploration in the direction of the therapist's hypothesis. As the client does not take this direction, in her second intervention, the therapist formulates the inferred emotion explicitly, thus showing more clearly that she ascribes to the client a motive or feeling that he did not address yet.

### Interpreting the Client's Way of Designing the Psychotherapeutic Relationship: Claiming an Analogy Between Agentive Self-Relationship and Interpersonal Relationship

In extract 3, the therapist draws an analogy between the topic of the client's story (self-control) and his interactional conduct (his attempt to control the therapeutic relationship).

Throughout the therapy, the client repeatedly reports about measures he takes in order to preserve his health. The therapist comments on one of these stories by ascribing to him that he acts “well-organized, well-reasoned and controlled” (082–084).

**Extract 3:** Therapy I_14_32:23-38:25.

078	TH	=äh äh sie sind ja: sehr bemÜht glaub ich auf VIElen Ebenen,=
		*erm erm you are y'know very eager on many levels*
079		=die: WEGzubauen;=
		*to discard*
080		=also sie sind ja gehen ja auch aktiv DRAN.=
		*I mean y'know you are y'know you actively*
081		= < < creaky> ja>,
		*yes*
082		(0.3) aber auch imme::r se:hr (0.1) organiSIERT;=
		*but always very well organized*
083		=überLEGT;=
		*reasoned*
084		=kontrolLIERT auch.
		*controlled as well*
085		(0.7)
086	CL	aber wisse sie wo des HERkommt;=
		*but d'you know where this comes from*
087		=weil ich viel im GLETscher gelernt hab.
		*because I have learnt much in the glacier*
(…)		
144		und die (.) die h Öchste äh äh konzentrationsübunge (0.3) sind
		beim FELsenklette[rn,]
		*and the highest erm erm concentration exercises are when climbing rock mountains*
145	TH	[hm]_HM,
146		(1.3)
147	TH	ne ich glaub sie haben VIEle sachen in ihrem lEben gemacht,=
		*well I guess you did many things in your life*
148		=wo sie sehr viel kontrOlle: (0.2) und konzentration dfür geBRAUCHT haben.
		*where you needed very much control and concentration*
149	CL	(0.2) ja:,
		*yes*
150	TH	(und ja) das hat ihnen sehr sehr viel geNUTZT,=
		*and yes this has helped you very very much*
151		=in vielen situationen in ihrem leben KLAR zu kommen.
		*to come to terms with many situations in your life*
152	CL	(0.1) hm;
153	TH	sowohl im SPO:RT;=
		*both in sports*
154		=als ouch in ihrer A:Rbeit;=
		*and in your work as well*
155		=wo sie auch va (.) viel [verant]wortung überNOMmen ha:ben;
		*where you also have assumed much responsibility*
156	CL	[ja,]
		*yes*
157	CL	ja,
		*yes*
158	TH	(0.5) und SO,
		*and so on*
159		°h (.) un ich glaube des-
		*and I guess this*
160		(1.8)
161	TH	des is ja in ihnen DRIN.
		*this is inside you y‘know*
162		(.) ((schmatzt))°h un ich hab mAnchmal das gefühl dass sie das
		auch hier bei UNS (0.34) sehr machen.
		*((lipsmack)) and sometimes I have the feeling that you do this*
		*here with us very much as well*
163		(.) also dass sie sehr
		kontrolLIE[ren;=
		*I mean that you control very much*
164		=was][sie e]rZÄ :Hle[n;]
		*what you tell*
165	CL	[wenn ich][mit ihnen] [S]PRECH;
		*when I talk with you*
166	TH	(0.1) ja:,
		*yes*
167		(0.9)
168	TH	äh:m; (0.2) un mi:r (1.1) mich auch nur zu_nem gewissen gra::d
		(.) ihnen HELfen lassen oder so.=
		*erm and (that you) let me (1.1) me help you only to certain degree or so as well*
169		=also so sehr vorgeben_n THEma?
		*I mean like you predefine a topic*
170		(0.7)
171	TH	un mir so_n bIsschen was erLAUben?
		*and (you) allow me a little bit*
172		(1.0)
173	TH	a::be::r da ouch_ne kontROLle drin lassen,=
		*but you still keep some control*
174		=wie viel mir pasSIERen darf in der therapie.=
		*how much may happen to me (=client) in the therapy*
175		=und wie viel geSAGT werd[en darf.]
		*and how much may be told*
177	CL	[ich WÜNsch m]ir
		*I wish*
178		ich WÜNsch mir
		*I wish*
179		(0.6)
180	CL	vielleicht sehen sie das SO:,
		*perhaps you see it this way*
181		(0.6)
182	CL	wenn sie des so empFINde,=
		*when you feel it this way*
183	CL	=Isch es [aber] in mir net SO.
		*but in me it is not this way*
184	TH	[hm–]
185	TH	(0.1) hm_HM,
186	CL	ich WÜNsch mir dass sie mir helfe.
		*I wish that you help me*
187	TH	(0.2) hm_HM,
188	CL	(0.2) ich würd
		*I would*
189		ich wünsch mir (0.2) dass ich mich richtig schön auf sie EINlasse kann.
		*I wish that I can really fully engage with you*
(…)		
267	TH	hm des war auch gar nich als VORwurf von mir
		[gemeint;=
		*mmh I didn't mean it as a reproach at all*
268	CL	[ne NE;]
		*no no*
269	TH	=des war einfach] so als_ne beSCHREIbung;
		*it was simply like as a description*
270	TH	^°^h (.) [und sie] haben das jetz so geSA:GT,=
		*and you now have said it this way*
271	CL	[ja,]
		*yes*
272	TH	=und ich glaube ihnen ihren WUNSCH auch;=
		*and I believe you that this is your wish*
273		=also [((creak))] (0.7) müssen sich keine SORgen machen;=
		*so (you) don't have to worry*
274	CL	[hm_HM;]
275	TH	=dass ich ihnen (.) das nich ABnehmen würde;
		*that I don't buy this from you*
276	CL	^°^h sie haben des
		*you have this*
277	TH	(0.2) da klang wie so_n ABer mit (0.1) in ihrem satz.
		*it sounded like in your sentence a “but” was included*
278		(.) als sie gesagt haben sie WÜNschen sich das?
		*when you said you wish this*
279		(1.1)
280	TH	aber es gibt
		*but there is*
281		(.) als gäbe_s Irgendwas was auch daGEgen spricht.
		*as if there was something which stood against it as well*
282		(1.3)
283	CL	[hm–]
284	TH	gib[t_s da ir]gend_nen WIDerstand;=
		*is there any resistance*
285		=oder_ne AN:gst,=
		*or a fear*
287		=auch davor vielleicht was dann: (0.1) pasSIEren könnte;=
		*also about what perhaps then could happen*
286		=was dann RAUSkommen könnte;
		*what could come out then*
288		(1.5)
289	TH	was passiert wenn sie die kontROLle auf (.) geben,
		*what happens if you give up control*
290		(0.6)
291	CL	WISse sie;
		*look*
292		(1.6)
293	CL	(doch/durch) des dass ich
		*(but/by) that that I*
294		(0.3)
295	CL	((clears throat)) ich kann_s immer (.) nur (.) wiederHOle,
		*I can always only repeat*

#### The Client's Story: Autobiographical Self-Positioning as Acting With Care and Concentration

The client seems to receive the therapist's comment about him acting well-organized, reasonable, and controlled (082–084) as praise. He responds by telling a story about how he learned to act with great care and concentration when climbing in the glaciers (086–144). This story expands a series of autobiographical stories in which he portrays himself as exerting agency (080) in a cautious and well-planned manner (082–084).

#### The Therapist's Response: Shift to the Way in Which the Client Manages the Therapeutic Relationship

The therapist first confirms the client's autobiographical identity claims (147–161), but then shifts to his behavior in the therapy (162–176).

Re-using her notion of “control” as a leitmotif of her interpretation of the client's self (084, 148, 163, 173), the therapist confirms that the client has come to terms successfully with many situations in his life (148–155) by “very much control and concentration” (148). She concludes that this strategy is deeply rooted in the client's self (159–161). She continues by drawing an analogy between the way in which the client controls himself and his life outside of the therapy with his verbal behavior in the therapy and the way he manages the therapeutic relationship (162–175). She claims that the client controls topics and the ways in which they are talked about (164, 169, 175) in a way that limits the scope of therapeutic help from her (168, 176).

#### Ensuing Negotiation: Denial of Limited Openness vs. Claim to Resistance Out of Fear

The client insists on the wish that the therapist helps him (177–186) and affirms that he wants to engage fully with the therapist (188–189). Yet, while accepting the authenticity of the client's wish (267–275), the therapist reframes her assumption that the client resists against giving up control (277–289).

In response to the therapist's ascription that the client controls and limits topics (162–176), the client claims his wish to be helped by the therapist and to engage fully with the therapist (177–189). Therapist and client thus express oppositional views on the present, interactive self of the client concerning his aim for interactional control and openness. The client's lengthy account of his wish to open up fully to the therapist, which is continued in the elided part of the excerpt (190–266), is responded to by the therapist by an overt action ascription: she denies that her prior action was to be understood as a “reproach,” but recategorizes it as a “description” of the client's way of interacting in the therapy (267–269). She adds that she believes in the seriousness of his wish to be helped by her (272–275). Both concessions are designed to discard the impression that seemed to underlie the client's affirmations, namely, that the therapist attacks the client's moral self in not believing in his authentic and unrestrained engagement in the therapeutic relationship. As before (078–084, 147–161), the therapist takes care to explicitly confirm the client's own positive identity claims (267–273). Yet, these affiliative affirmations are only preliminaries to insisting on her prior ascription that he is afraid of losing control (277–291). She first claims that the client “sounds” (277) as if his talk included an unspoken concessive part, a “but” (277). This time, the therapist not only reiterates the ascription of “control” to the client (289) but also adds more far-reaching motivational ascriptions, asking whether there is “resistance” (284) or “fear” (285) in the client against giving up control. Although the therapist's interpretation is couched in terms of an interrogative, her insistence on the topic and the evidence from the sound of his talk clearly show that she assumes the client to exhibit resistance. Starting in 291 (and continuing beyond the extract), the client reaffirms that he fully engages in the therapy, while conceding that he may not seem to be as “relaxed” as the therapist might want him to be.

##### Self- and other-positioning in extract 3

The client positions his autobiographical, told self as acting cautiously, planfully, and with a large amount of concentration, i.e., as a person with a high degree of agency. In response to the therapist's challenging ascriptions, he explicitly positions his present, interactive self as engaging fully with the therapy in order to get optimal help.

The therapist fully affiliates with the client's explicit self-positioning, which she explicitly confirms. She uses the psychoanalytic technique of proving the lingering relevance of past experiences for the client's present self by claiming that the client re-enacts biographically entrenched patterns of interaction in the therapy (Levy, 1998). By this, the therapist shifts from the level of the client's autobiographical self-positioning to the client's interactive, present self by way of analogy. The client is other-positioned as acting in a way that is unnoticed by himself and contradicts his explicit claims. The therapist makes a distinction between believing the client's intentions (not to control and restrict his performance in therapy) and her assumption that the client does not act according to what he claims, thus introducing the distinction between a conscious and an unconscious self of the client as an interactionally relevant reality.

Over the extended negotiation about the relevance of “control” to the client's actions beyond and within therapy, therapist and client do not reach an agreement about how to conceive of the client's interactive self. While both agree on the client's explicit self-ascriptions, the therapist claims that there are additional unconscious motives, which run counter to the intentions of the conscious self. Yet, the therapist unpacks these motivational ascriptions only after the client has not accepted her ascription of a controlling behavior in the therapeutic context and has not engaged in self-exploration concerning the possible motif that the therapist has ascribed to the client.

### Observing Non-verbal Conduct: Focusing on an Emotion and Shifting its Object

In extract 4, the therapist focuses on an emotion (sadness) that the client expresses mainly non-verbally and he shifts the object of the emotion to a more self-related concern.

Extracts 4 and 5 come from the second therapy with a senior male therapist and a female client, who suffers from psychogenetic seizures. The client has just told her boyfriend that she breaks up their relation. She talks about her concern that, after they separated, she won't be able to help him anymore.

**Extract 4:** Therapy C_20_20:34-22:50.

01	CL	also ich BRAUCH das nich dass ich irgendwem (0.2) so HELfe;=s
		*I mean I don‘t need it that I help anybody*
02		(0.6)
03	CL	eher darum dass (0.6) äh:m (2.3) ^°^h mein HERZ dabei (0.7) extrems wEh tut;
		*rather it's about that erm my heart is aching extremely*
04	TH	hm_HM;
05	CL	bei dem gedAnken dass ich ihm dann
		*thinking that then I him*
06		(0.6)
07	CL	ähm:
		*erm*
08		(0.4)
09	CL	ja,=nicht mehr n:: ja;=
		*well no more/ well*
10		=ihn nicht AUF:fangen kann,
		*not being able to catch him*
11	TH	hm_[HM,]
12	CL	[nicht HELfen] kann,=einfach
		*not being able to help simply*
13		(0.4)
14	CL	dess: (0.1) er dass ihm halt nicht GUT geht.
		*that he that he just is not well*
15	TH	(0.1) hm_HM,
16		(0.8)
17	CL	dass dass er
		*that that he*
18		(1.2)
19	CL	ich könnte locker drauf verZICHten,
		*I could easily do without*
20	CL	((aborted laughter)) < < laughingly> ihm zu HELfen >;=aber
		*((laughs)) helping him but*
21		(0.8)
22	CL	[äh:m:]
		*erm*
23	TH	[HM_hm,]
24		(1.6)
25	CL	nur wenn_s ihm dann GUT geht.
		*only if he is well then*
26		(0.5)
27	TH	HM_hm;
28		^*^ (3.0) ^*^(3.5)^*^ (3.7) ^*^ (2.7) ^*^(1.5)^*^ (6.5) ^*^
	Cl	^*^nods 3x^*^^*^nods 2x^*^crisps lips^*^^*^crisps/bites lips^*^
29		^*^ (5.7) ^*^
	Cl	^*^eyes fill with tears^*^
30	TH	((lipsmack)) (.) so_n bisschen traurig macht sie das SCHON dieser gedanke.=ne?
		*it makes you kinda little bit sad though this thought*
31		(0.5)
32	CL	((lipsmack)) < < f> ja:;>=
		*yes*
33		=^*^auf jeden FALL,>
		*definitely*
	cl	^*^smiles———->
34	CL	[jaha;]^*^
		*yes*
	cl	——–>^*^
35	TH	ja [da]ss ihre wege jetzt auseinANder gehen,=
		*well that you will be going separate ways now*
36		=dass sie (0.76) ihm danach nich (0.46) nich mehr
		*that you him afterwards no no more*
37		(0.3)
38	TH	((lipsmack)) < < creaky> ja >.
		*yes*
39	TH	((lipsmack)) HELfen können.
		*will be able to help*
40	CL	(0.2) ja.
		*yes*
41		(6.5)
42	CL	^°^h da muss ich halt wIrklich auf mich AUFpassen,
		*I really just have to take care of myself*
43		(0.3)
44	CL	wenn ich dann zu HAUse bin,
		*when I will be at home then*
45		(0.3)
46	CL	^°^hhh dass ähm: (1.8) ja,=ich dann nich so (2.9) in diesen
		(0.8) in diese TRAUer;=
		*that erm well I then don‘t drown so much in this in this sadness*
47		=schmErz oder so was da (0.5) hiNEINverfalle (.)
		*pain or kinda something of that kind*
		[sozu]sagen;=also ^°^h
		*so to say I mean*
48	TH	[hm;]
49		(1.9)
50	CL	dass mich das nicht zu:: (0.3) se:hr EINnimmt;
		*that it doesn't absorb me too much*
51		(0.8)
52	TH	hm_hm;
53		(0.8)
54	CL	sondern dass ich halt auf MICH (.) natürlich gUcke;
		*but of course that I just look after myself*
55	CL	(.) auf mich AUFpasse,
		*take care of myself*
56	TH	hm_HM;
57		(5.3)
58	CL	mhja:hh,
		*myes*
59		(1.4)
60	TH	^°^h des is ja sicher WICHtig;
		*this is certainly important*
61		(0.5)
62	TH	andererseits ein bisschen (0.75) auch die traurigkeit spÜren
		^°^h hilft vielleicht (.) dann auch (.) sich innerlich (1.3)
		dann auch noch mehr (0.3) zu verABschieden.
		*on the other hand a little bit also to feel the sadness perhaps*
		*then also helps to say fare well still more mentally*
63		(0.8)
64	CL	ja,=auf jeden FALL;
		*yes definitely*
65		(0.8)
66	TH	< < p> gehört halt daZU,>
		*y'know it's part of it*
67		(0.3)
68	CL	< < creaky, p> ja>;
		*yes*

#### The Client's Story: Concern About Her Boyfriend (01–25)

The client had told about her boyfriend's eating disorders and his problems to come to terms with his life. She tells that she feels bad when thinking that she won't be able to help him anymore in the future after they split. In the lengthy pause that emerges after her account (28–29), the client nods several times and looks away from the therapist; she crisps and bites her lips; her eyes begin to fill with tears.

#### The Therapist's Response: Focus on the Client's Emotion (30–39)

The therapist provides an interpretation that assigns an explicit emotional interpretation to the client's non-verbal conduct (cf. Muntigl and Horvath, 2014): “it makes you kinda little bit sad though this thought” (30). The modal particle “schon” indexes a concession that the therapist expects the client to make, thus showing that he conceives his interpretation to be at least partially different from her explicit self-presentation (30). The client confirms without reserve, slightly smiling (32–34). However, the therapist continues his turn by explicitly adding a formulation of the object of her sadness, namely, the separation from the boyfriend (35), which is different from what the client had talked about before, i.e., the inability to support her boyfriend anymore (see 09–12). Still, the therapist then realigns with the client's prior focus of not being able to help anymore (36–39).

#### Ensuing Negotiation: Joint Shift to Client's Self-Related Emotions and Need for Coping

Taking up the therapist's ascription of sadness (“traurig,” 31 → “traUer,” 46), the client elaborates on it by claiming that she will have to take care of herself by not letting herself drown in this emotion (42–55). The therapist explicitly agrees (60), but then claims that the client will need to face the sadness about the end of the relationship in order to be able to say farewell and psychologically cope with it more comprehensively (62). Both agree (64–68).

##### Self- and other-positioning in extract 4

The client first explicitly positions herself as being distressed by not being able to comply with the exigencies of her moral ideal self concerning the support she should provide her boyfriend.

The therapist does not question the explicit self-positioning of the client. Rather, he gradually shifts the focus away from the boyfriend and the client's moral self to her own emotions (Peräkylä, 2008; Voutilainen et al., 2010) caused by the experience of the split-up and the need to cope with them properly. This change is managed by attending to the client's performative, non-verbal displays and by topicalizing their emotional content.

The therapist's shift from the client's explicit self-positioning as being concerned about the loss of her ability to help her boyfriend to the emotion of sadness implies a shift in focus from the other-related concern about the boyfriend to the client's emotional self. This combines with a shift of the object of the emotion, namely, from the loss of the ability to help to the loss of the relationship itself. This shift paves the way for the therapist's permission to allow for her sadness sufficiently in order to be able to cope with the separation from the boyfriend. The therapist's shift to the client's performative self is thus used as a cue to enhanced emotional self-exploration by the client, on which the therapist builds his recommendation.

The client aligns with the therapist's shift to her performative emotional self and affiliates with his statements. The shift in focus establishes the common ground between therapist and client about the client's emotional state that is needed as a basis for the intelligibility and acceptability of the therapist's recommendation.

### Summarizing Impressions From Client's Talk: Challenging the Authenticity of the Performance

In extract 5 from an earlier session, the therapist seemingly gives just a summary of the client's prior talk, which, however, can be heard as challenge building on the impression that the client interactional performance conveys. The client had talked for the first 11 min about her current life situation (preparing university exams, leisure-time activities, boyfriend, and plans to move to another town). She stated that she did not suffer from seizures recently, but reported eating problems, which, however, she claimed to be under control of “her head” (01–06).

**Extract 5:** C_2: 11:10-12.40.

01	CL	ja.=aber darauf (0.31) schaltet sich mein kOpf meistens dann ganz gut EIN.
		*but then mostly my head intervenes properly*
02	TH	HM[hm,]
03	CL	[und der sa]gt (.) hier (.) trotzdem,
		*and it says nevertheless*
04	TH	(0.36) HM_hm,
05	CL	< < p> iss> trotzdem [(so)]
		*eat nevertheless so*
06	TH	[sodass das gewicht] jetzt nicht
		zu stark
		nach UNten gegangen [ist] bis jetzt.
		*so that your weight hasn't decreased too much until now*
07	CL	[nein.]
		*no*
08	TH	oder nicht GEhen (wird).
		*or (will) not decrease*
09	TH	^°^h
10		(1.39)
11	TH	((lipsmack)) ja,
		*yes*
12	TH	(0.61) ^°^h also eigentlich wenn man ihnen so ZUhört,=
		*so actually if one listens to you*
13		=muss man SAgen,
		*one must say*
14	TH	^°^h klingt es so dass sie jetzt im AUgenblick mit a (0.2)
		*it sounds as if now at the moment with*
15		mit AUSnahme eigentlich dieser (0.44) ^°^hhh körperlichen beschwerden,
		*with the exception of your bodily problems*
16		(.) ihr leben eigentlich so ganz gut im GRIFF haben.=Oder?
		*you can cope with your life quite well, right?*
17		(0.25)
18	TH	hm (.) klingt SO.=Oder?
		*sounds like that right?*
19	TH	würden sie,
		*would you*
20		wie sehen SIE das;
		*how do you see it?*
21	CL	^°^hhh
22	TH	äh:m:
23		(1.41)
24	CL	jein,
		*yes-no*
25	CL	((laughs))
26	TH	hm_HM?
27		(0.37)
28	CL	äh:m: (.) bei mir ist es SO dass ich das ähm: (1.71)
		*erm with me it is this way that I am able erm*
29		gut nach AUßen;
		*well to the outside*
30		((laughs)) so:;
		*like*
31	TH	hmHM?
32	CL	(0.4) ähm DARstellen kann [glaub ich auch.]
		*erm to present I guess*
33	TH	[hm_HM,]
34	TH	^°^h ah JA.
		*oh I see*
35	CL	also ich (0.25) erTAPpe mich auch;
		*so I catch myself as well*
36	CL	(.) !SEIT! diesem klinikaufenthalt hab ich das geMERKT,
		*since this stay at the hospital I have realized*
37	CL	^°^hh äh:m: weil mir des auch da ein therapeut geSAGT hat,
		*erm because there a therapist also told me*
38		(0.43)
39	CL	dass (.) ich ä:hm auf VIEles immer schön gleich wegLÄCHel?
		*that I erm in response to many things I always just smile them away*
40	TH	hm_HM,
41	CL	(0.28) ah:m: und (0.98) wenn ich so EINS zwei mal über dinge
		geSPROchen hab;
		*erm and when I like once or twice talked about things*
42		(0.3)
43	CL	dass ich dann auch relativ neutRAL.
		*that I reported relatively neutral then as well*
44		(1.04)
45	TH	hm_[HM,]
46	CL	[DAR]stell:e;

#### The Client's Story: Presentation of a Rational and Trouble-Free Self (Until 05)

The client tells about various domains of her life, mostly smilingly. She does not mention major problems and presents herself as a well-organized and goal-oriented person by her account. Her health problems are only addressed in response to an inquiry by the therapist; yet, they are portrayed by the client as being under her control.

#### The Therapist's Response: Challenging the Impression the Client Gives by Mirroring (06–20)

The therapist completes and thereby confirms the client's story, stating that she manages to control her weight appropriately (06–08). He summarizes the impression created by the client's account with “you can cope with your life quite well” (16). This summary is framed by verbs of perception (12, 14, 18) as how the client “sounds” when “listening” to her. In particular, the concluding repeat “klingt SO. = Oder?,” “sounds like that, right?” (18) can be heard as a challenge, because again the perceptual impression that the client's story conveys, but not its truth, is highlighted. The tags *oder?* (“right,” 16, 18; see König, 2020) and the following questions (19–20) explicitly ask the client to take a stance on this impression, thus implicitly calling, or at least allowing, for the client's self-repair.

#### Ensuing Negotiation: Client Admits Insincerity

With the ambiguous response token “jein” (a blend of *yes* and *no*), the client indexes that no straightforward answer is possible and projects a complex elaboration (Bücker, 2013). The client avows that she is able to give a favorable, unproblematic impression “to the outside” (28–32), thus letting infer that this does not correspond to how she actually feels. When saying that she “catches herself” (35) when “smiling away many things” (39), she adumbrates that this is a habitual way of presenting herself that she herself often is not aware of, but was only averted to by a former therapist (37).

##### Self- and other-positioning in extract 5

The client initially positions herself as a rational, goal-oriented person, who can cope with the (minor) troubles she faces. The therapist confirms this positioning, yet by stressing its perceptual nature and explicitly asking for the client's take on this impression, he implicitly leaves room for doubt and indexes the need for deeper elaboration concerning the validity of the client's self-positioning. The therapist does not explicitly other-position the client. In response, the client avows a lack of sincerity in her self-presentation, distinguishing a habitual facade of unproblematicity for others from the real self^3^, which, however, she does not elaborate on in the context of the extract.

## Conclusion

In Western culture, there is a deep-rooted assumption that subjects have privileged access to their own self (Heritage, 2011; Gertler, 2020). This assumption is embodied in most interaction types in the preference not to question subjective experience and self-related statements, but instead to affiliate with them and show empathy. Yet, the rationale of almost every psychotherapy includes to cause psychological change by altering clients' self-perceptions, self-ascriptions, and understandings of motives and goals by the therapist's interventions. This includes questioning the client's epistemic authority on their own self, which is a sensitive move that has to be carefully considered each time anew.

One way to question the client's authority concerning their self is to shift from the client's focus on their autobiographical, told self of which they are conscious to their performative self, which they enact in the interaction with the therapist. This latter self usually is rather, in terms of Goffman (1959), “given off” than part of what the client intentionally conveys about their self. In psychoanalytic terms, addressing the performative self thus often means to address unconscious and sometimes conflictual aspects of the client's personality, feelings, and motives. Prior research on interpretations has shown that they regularly include ascribing emotions to the client that they have not explicitly addressed or even seemed to hide (e.g., Vehviläinen, 2003; Peräkylä, 2008; Voutilainen et al., 2010; Muntigl and Horvath, 2014). Our analyses show that shifts to the performative level of client's conduct therapists' interpretations can still address other facets of the client's self. In particular, they can concern motives for their interactional conduct that the client did not address in their talk, claims about how the client designs the current interaction with the therapist, a shift in causes or objects that are seen as causes for the client's emotions, and challenges of the authenticity of the client's self-positioning, again pointing to unavowed motives and emotions of the client. The shift to topicalizing such performative aspects of the client's conduct amounts to a severe face threat in at least two ways: epistemically, the client is treated as not being (fully) authoritative on their own self; morally, the adequacy of the client's behavior, their goals, and motives are put into question. Thus, psychotherapists are faced with the dilemma to get clients to conceive of their selves in new and often oppositive ways, but yet to respect the clients' face.

In the data, we could see that therapists deal with this dilemma by a particular design of the interpretations that address the performative self of the client as the therapist perceives it *in situ*. Therapists produce lengthy multi-unit turns that start with a display of understanding and empathy with the client that explicitly ratify the client's perspective. Only after this do they turn to a competing perspective that the client does not seem to be aware of, but that is treated as equally or even more important, ascribing behaviors, motives, and feelings that are different from the ones the client has addressed (extracts 2–5) or that serve as a motivational explanation (extract 1). This competing perspective is not just posited, but argumentatively backed with reference to the client's own prior talk (Weiste et al., 2015), to a common ground that has been built earlier in the psychotherapy, general world knowledge, and the therapist's own imaginations concerning the told episodes. Thus, the interpretation is not just delivered as a unilateral observation from a more authoritative, expert position, but therapists try to ground it intersubjectively in joint observation of the client's talk and behavior and their shared interactional history. Furthermore, the therapist's interpretation is introduced in a more or less tentative, hypothetical way (see Stukenbrock et al. submitted), which invites the client to self-explore further in the direction of the self-aspects that the interpretation points at. This observation corresponds to current conceptualizations of psychoanalytic interpretation (Heimann, 2016; Civitarese, 2020) that stress its intersubjective and relational aspects.

Yet, this complex and careful design of the therapist's shift to the client's performative self does not always cause clients to align with it, agree with the therapist's interpretation, and indulge in enhanced self-exploration (as in extracts 1, 4, and 5). They may resist (extract 3) or confirm only partially or in passing without entering into a more detailed exploration of the interpretive perspective that the therapist offers (extract 2). In addition, as extract 1 has shown, there may even be a deep misunderstanding between client and therapist in the sense that the client overtly agrees with the therapist, while misconstruing the therapist's action and assessment.

We found that the therapists' shift to the performative self involves focusing on the client's feelings and motives, but less on categorical psychological or moral identity claims (e.g., strength, rationality, honesty), which are focal in clients' stories. Thus, different facets of the self matter to different participants. While prior research on psychotherapy has shown that therapists' interpretations attend in particular to emotions that the client did not address, the positioning perspective adopted in this paper enlarges the picture of how therapists attend to latent psychological aspects. Psychodynamic therapists are more generally sensitive to the here-and-now performance of the client, which importantly includes their ways to conduct the interaction with the therapist, their bodily displays and motives that their discursive actions make available for the therapist. In this way, therapists attend to a larger notion of the client's self that transcends autobiographically based narrative representations in favor of the vision of a performative self that reveals itself in the ways it acts *in situ*.

Sequential analysis of the negotiations between client and therapist following the therapist's shift to the performative self show that such shifts promote the therapeutical agenda by inviting the client's self-exploration (cf. Peräkylä, 2010). Therapists do not necessarily expect clients to confirm straight away the interpretations that they tentatively offer about the client's self. Rather, they use them to elicit and deepen the client's self-exploration, which does not always have to follow closely along the lines of what the interpretation has suggested. Rather, the interpretation can be treated by both parties as a starting point for exploring a better understanding of the client that is to be worked out collaboratively. However, if the client resists to this elicitation, therapists insist on their interpretation, often couching it in more definite and more saturated terms (Will, 2016), making it less tentative (as in extract 3). The shift in positioning levels thus seems to have a preparatory function for promoting the therapeutic agenda and for engendering therapeutic insights, which build immediately on what is intersubjectively observable in the client's multimodal conduct.

## Data Availability Statement

All datasets presented in this study are included in the article.

## Ethics Statement

The studies involving human participants were reviewed and approved by Ethik-Kommission der Albert-Ludwigs-Universität Freiburg. The patients/participants provided their written informed consent to participate in this study. Written informed consent was obtained from the individual(s) for the publication of any potentially identifiable images or data included in this article.

## Author Contributions

All authors have conducted the analyses presented. AD has written the paper. CS was responsible for data collection. All authors contributed to the article and approved the submitted version.

## Conflict of Interest

The authors declare that the research was conducted in the absence of any commercial or financial relationships that could be construed as a potential conflict of interest.

## Footnotes

^1^Transcript headers designate the therapy series (I or C), number of session, and beginning and end time of the extract within the session.

^2^See Stukenbrock et al. (submitted) for the use of meta-perceptual verbs in therapists' interpretations.

^3^The client's distinction between a publicly displayed and a real self is reminiscent of Reich's psychoanalytic account of the “facade” (Reich, 1933) and Goffman's socio-psychological account of self-presentation (Goffman, 1959).

## Appendix

**Appendix A:** Transcription Conventions GAT 2 (Selting et al., 2011).

[ ]	overlap and simultaneous talk
=	immediate continuation with a new turn or segment, latching
°h/h°	in-/outbreaths of ~0.2–0.5 s duration
°hh/hh°	in-/outbreaths of ~0.5–0.8 s duration
(.)	micro pause, estimated, up to 0.2 s duration
(0.5)	measured pause
and_uh	cliticizations of units
uh, uhm, etc.	hesitation markers, so-called “filled pauses”
:	lengthening, by about 0.2–0.5 s
::	lengthening, by about 0.5–0.8 s
((laughs))	description of laughter and crying
< < laughing>>	comment on speech delivery with indication of scope
SYLlable	focal accent
sYllable	secondary accent
!SYL!lable	extra strong accent
?	rising to high
,	rising to mid
–	level intonation
;	falling to mid
.	falling to low
<<p>>	piano, soft
<<f>>	forte, loud
<<decr>	decrescendo, becoming softer
(may i)	assumed wording

**Appendix B:** Multimodal Transcription Conventions (Mondada, 2018).

^**^	Gestures and descriptions of embodied actions are delimited between two identical symbols (one symbol per participant) and synchronized with correspondent stretches of talk.
^*^−−−>	The action described continues across subsequent lines.
−−−−>^*^	until the same symbol is reached.
—-	Action-apex is reached and maintained.
